# Platinum-Induced Peripheral Neuropathy (PIPN): ROS-Related Mechanism, Therapeutic Agents, and Nanosystems

**DOI:** 10.3389/fmolb.2021.770808

**Published:** 2021-11-24

**Authors:** Xi Hu, Zhijie Jiang, Longyu Teng, Hongyu Yang, Dongsheng Hong, Dongsheng Zheng, Qingwei Zhao

**Affiliations:** ^1^ Department of Clinical Pharmacy, The First Affiliated Hospital, Zhejiang University School of Medicine, Hangzhou, China; ^2^ Zhejiang Provincial Key Laboratory for Drug Evaluation and Clinical Research, The First Affiliated Hospital, Zhejiang University School of Medicine, Hangzhou, China

**Keywords:** platinum, peripheral neuropathy, reactive oxygen species, mechanism, therapeutic agents, nanosystems

## Abstract

Platinum (Pt) drugs (e.g., oxaliplatin, cisplatin) are applied in the clinic worldwide for the treatment of various cancers. However, platinum-induced peripheral neuropathy (PIPN) caused by the accumulation of Pt in the peripheral nervous system limits the clinical application, whose prevention and treatment are still a huge challenge. To date, Pt-induced reactive oxygen species (ROS) generation has been studied as one of the primary mechanisms of PIPN, whose downregulation would be feasible to relieve PIPN. This review will discuss ROS-related PIPN mechanisms including Pt accumulation in the dorsal root ganglia (DRG), ROS generation, and cellular regulation. Based on them, some antioxidant therapeutic drugs will be summarized in detail to alleviate the Pt-induced ROS overproduction. More importantly, we focus on the cutting-edge nanotechnology in view of ROS-related PIPN mechanisms and will discuss the rational fabrication of tailor-made nanosystems for efficiently preventing and treating PIPN. Last, the future prospects and potential breakthroughs of these anti-ROS agents and nanosystems will be briefly discussed.

## Introduction

Platinum (Pt) drugs, such as cisplatin (CP), oxaliplatin (OXA), and carboplatin, are commonly used in the treatment of colorectal cancer (Saltz et al., 2008), gastric cancer (Shen Q.-D. et al., 2018), breast cancer (von Minckwitz et al., 2014), and lung cancer (Ferrara et al., 2021) in combination with other chemotherapy drugs. The antitumor mechanisms of Pt drugs are mainly DNA damage and enhanced reactive oxygen species (ROS) generation (Rottenberg et al., 2021). The excess ROS, including superoxide anion (·O_2_^−^), singlet oxygen (^1^O_2_), hydrogen peroxide (H_2_O_2_), hydroxyl radical (·OH), and so on, would induce oxidative damage to biomolecules (e.g., proteins, lipids, and DNA) and, thus, lead to severe cellular damage (Fasnacht and Polacek, 2021). However, the toxic effects of Pt drugs affect the quality of life of patients and cause reduction or discontinuation. Among the toxic effects, platinum-induced peripheral neuropathy (PIPN) has the symptoms including paresthesia, extremities pain, and cold sensitivity. Herein, PIPN can be divided into acute peripheral neuropathy and chronic peripheral neuropathy. Acute peripheral neuropathy that usually occurs in a few hours after Pt drug administration is easily induced by the exposure to cold temperature, whereas chronic peripheral neuropathy tends to appear upon reaching a certain cumulative dose [e.g., OXA of 780 mg/m^2^ (Rothenberg et al., 2003), CP of 350 mg/m^2^ (Krarup-Hansen et al., 2007)] and can be characterized by sensory neurotoxicity (e.g., sensation loss and changes) due to the accumulation of Pt drugs at the peripheral nervous system (Cersosimo, 2005). Notably, the prevention and treatment of PIPN is an extremely urgent issue to be addressed in patients treated with Pt-based chemotherapy.

To solve the challenge, numerous researchers devote to exploring the mechanism of PIPN, which are principally implicated in transporter overexpression (Sprowl et al., 2013), ROS upregulation (Leo et al., 2020), ion channel dysfunction (Descoeur et al., 2011), transient receptor potential (TRP) overexpression (Chukyo et al., 2018), inflammatory response (Janes et al., 2015), and so on. It is noteworthy that Pt-induced ROS production impairs antioxidant enzymes [e.g., superoxide dismutase (SOD) and catalase (CAT)] to trigger oxidative stress (Khasabova et al., 2019), which participates in the onset and progression of both acute and chronic PIPN. Moreover, the Pt-induced ROS production further disturbs the normal function of DNA, mitochondria, microtubule, ion channels, and biomolecules, thus, inducing neuroinflammation, demyelination, and neuronal apoptosis in the process of PIPN (Lim et al., 2010; Areti et al., 2014; Bas et al., 2019; Stanford et al., 2019). Therefore, ROS is a double-edged sword, which can bring us antitumor therapeutic benefits and toxic effects. Herein, how to simultaneously attenuate PIPN and meanwhile maintain antitumor efficacy by regulating the ROS level in normal tissues and tumor tissues deserves to be thoroughly addressed.

Currently, some antioxidant agents have been utilized to treat PIPN in clinical trials and animal studies by relieving the Pt-induced ROS upregulation. Moreover, it is noteworthy that nanotechnology offers immense potential in preventing and alleviating PIPN via two main strategies: 1) enhancing the targeting accumulation and/or activity of Pt drugs at tumor tissues while decreasing the off-target effects and 2) specific delivery of antioxidant agents toward the peripheral nervous system. Herein, this review will discuss the ROS-related mechanism and feasible therapeutic drugs for PIPN. More importantly, PIPN-tailored drug delivery nanosystems have been elaborated and discussed by utilizing the cutting-edge nanotechnologies. Last, the future prospects and potential breakthroughs of these anti-ROS agents and nanosystems will be briefly discussed.

## Reactive oxygen species-related mechanism of platinum-induced peripheral neuropathy

**FIGURE 1:**
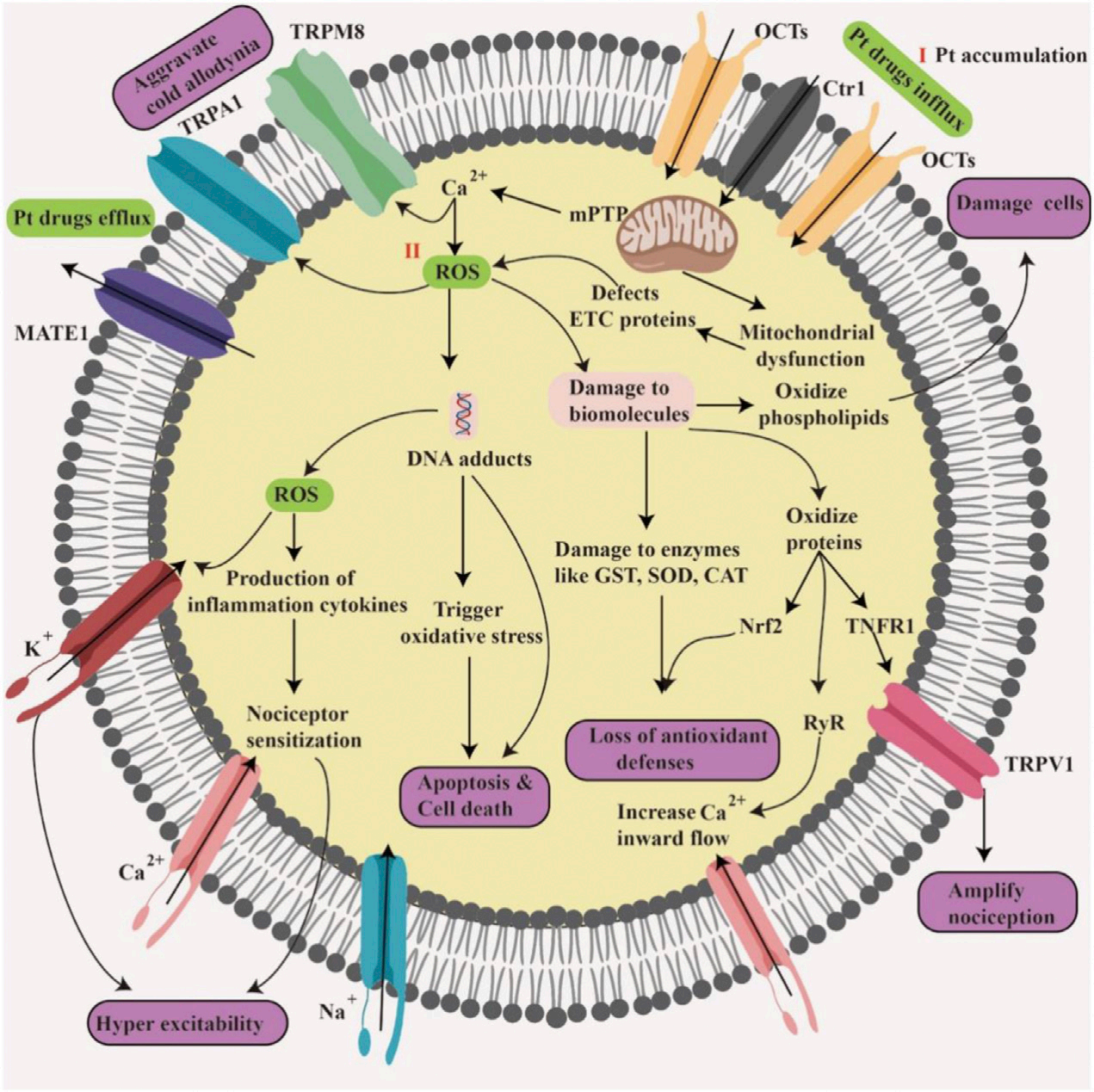
Reactive oxygen species (ROS)-related mechanism of platinum-induced peripheral neuropathy (PIPN). I. Pt drug accumulation is mediated by the uptake via OCTs and Ctr1, and efflux via multidrug and toxin extrusion protein 1 (MATE1). II. Pt drugs cause mitochondrial dysfunction and oxidative stress, leading to the imbalance in the endogenous ROS and cellular antioxidant systems. Moreover, Pt-induced ROS result in the damage to biomolecules, such as phospholipids and proteins [e.g., ryanodine receptor (RyR), NF-E2-related factor 2 (Nrf2), and tumor necrosis factor receptor 1 (TNFR1)], downregulation of antioxidant enzymes [e.g., SOD, CAT, and glutathione-S-transferase (GST)], the activation of transient receptor potentials (TRP), the oxidization of ion channels (e.g., K^+^, Ca^2+^, and Na^+^), as well as the release of inflammatory cytokines, ultimately aggravating the progress of PIPN.

The dorsal root ganglia (DRG), which is composed of sensor cells, neurons, and effector cells, plays a pivotal role in the process of PIPN. Pt drugs have been reported to result in mitochondrial damage and oxidative stress once they accumulate at DRG (Podratz et al., 2017). The upregulated Pt transporters [e.g., organic cation transporters (OCTs), multidrug and toxin extrusion proteins (MATEs), and copper transporters 1/2 (Ctr1/2)] in DGR increase the accumulation of Pt drugs in peripheral nerves as the first step to induce PIPN (Sprowl et al., 2013; Fujita et al., 2019). Moreover, ROS also feed back to regulate functional residues of PIPN-related proteins, for instance, ion channels and transient receptor potential ankyrin 1 (TRPA1), whose abnormalities exacerbate Pt-induced cold allodynia (Chukyo et al., 2018; Argyriou et al., 2019). Overall, the ROS-related mechanism of PIPN could be elaborated from the following two aspects: 1) Pt accumulation in DRG and 2) ROS generation and cellular regulation (Figure 1).

### Platinum accumulation in dorsal root ganglia

The accumulation level of Pt drugs in DRG is 10–20 times higher than that in other nerve cells, which could be attributed to the high expression of Pt-related transporters (e.g., Ctr1/2, OCTs) in DRG (Trecarichi and Flatters, 2019).

Among these transport proteins, Ctr1, composed of three highly conserved methionine (Met)-rich motifs, is involved in the transport of CP as a channel-like transporter (Yonezawa and Inui, 2011). The first two Met-rich motifs and last Met-rich motif are located at the extracellular N terminus and the end of the second transmembrane structural domain, respectively. Wherein CP interacts with the accessible Met-rich motifs to form the [Pt(Met)Cl(NH_3_)_2_] intermediate, which induces a conformational change in Ctr1 and allows CP to pass laterally through the axis of the trimeric Ctr1 channel and move inward via the intermolecular sulfur–sulfur process exchange (Liang et al., 2009; Kuo et al., 2021). Besides, the cellular uptake of CP would be reduced by nearly 80% after downregulating the expression level of Ctr1 in cerevisiae strains and mouse cells (Ishida et al., 2002).

In addition, OCTs (e.g., OCT1 and OCT2), as the active transport proteins, mediate the endocytosis and efflux of organic cations driven by intramembrane negative potentials. Since OCTs-mediated cellular uptake is concentrative and much more active than the Ctr1 type (Liang et al., 2009; Yonezawa and Inui, 2011), OCTs are the key transporter to exacerbate the PIPN (Jong et al., 2011). Moreover, MATE1, driven by the secretion of cationic drugs with opposite H^+^-gradients, is involved in the efflux of Pt drugs as well as other endogenous or exogenous organic cations in the DRG (Fujita et al., 2019). In brief, the enhanced cross-cellular transport of Pt drugs is mediated by the uptake via OCTs and efflux via MATE1, respectively (Yang et al., 2021), in which OXA and CP have been found to be substrates of the human OCT family and the human MATE family (Yokoo et al., 2007).

In brief, Pt-associated transporter proteins determine the accumulation of Pt drugs in DRG and, thus, could be developed as therapeutic targets to prevent the development of PIPN.

### Reactive oxygen species generation and cellular regulation

Overloaded Pt drugs produce toxic ROS, which are strongly associated with PIPN (Umeno et al., 2017; Zajaczkowska et al., 2019). In detail, ROS are involved in disturbing antioxidant defenses, the mitochondria, microtubule, ion channels, and biomolecular functions, as well as inducing neuroinflammation, demyelination, and neuronal apoptosis in the process of PIPN (Areti et al., 2014).

Pt-induced ROS production easily breaks the balance of endogenous ROS and cellular antioxidant systems to trigger oxidative stress and mitochondrial dysfunction, resulting in the damage or loss of DRG cells containing abundant ROS-sensitive phospholipids (Khasabova et al., 2019). In addition, as for DRG cells, NF-E2-related factor 2 (Nrf2) that upregulates the expression of antioxidant enzymes (e.g., SOD, CAT, and glutathione-S-transferase (GST)) to eliminate oxidative stress, would be disrupted by accumulated Pt drugs (Dos Santos et al., 2021).

Regarding ion channels, TRP play a key role in aggravating cold abnormal pain, among which, TRPA1 and transient receptor potential melastatin 8 (TRPM8), as cold-sensitive receptors, could be activated by ROS (Shimizu et al., 2014; Miyake et al., 2016). In detail, ROS could activate TRPA1, via oxidizing cysteine residues (Andersson et al., 2008), and TRPM8, via increasing Ca^2+^ influx (Bas et al., 2019) and upregulating ADP-ribose of the mitochondria (Shimizu et al., 2014). Under oxidation conditions, TRPV1 can overexpress tumor necrosis factor receptor 1 (TNFR1) in DRG cells through a ROS-mediated signaling pathway, which could bind with proinflammatory cytokine tumor necrosis factor α (TNF-α) to increase inflammatory conditions and nociception (Ma et al., 2009; Westlund et al., 2010).

Some voltage-gated ion channels, namely, the potassium (K^+^), calcium (Ca^2+^), and sodium (Na^+^) channels, could also be oxidized by ROS to consequently affect DRG neuronal excitability and conduction. For example, when voltage-dependent potassium channels-1 (KVS-1) are oxidized, they conduct more current and thus, affect DRG neuronal output. When KCNB1 (a homolog of KVS-1) contacts with ROS, Src/JNK-meditated apoptotic program in mitochondria would be initiated to produce more ROS (Patel and Sesti, 2016). In addition, the ryanodine receptor (RyR), a well-established redox-sensitive Ca^2+^ channel, would open more readily and thus, leak Ca^2+^ after oxidation (Oda et al., 2015). ROS also induce the release of Ca^2+^ by activating inositol 1,4,5-trisphosphate receptors (IP3R) to open the permeability transition pore, thereby changing the permeability of mitochondria to further release more ROS as a vicious cycle (Csordas and Hajnoczky, 2009; Kiselyov and Muallem, 2016). Moreover, voltage-gated sodium (NaV) channels play a role in initiating action potentials in DRG neurons, whose surface modification affects neuronal electrical excitability and participate in the development of cold abnormal pain (Furgala-Wojas et al., 2020). For example, Pt drugs prolong Nav1.6 open times and increase persistent current in DRG neurons, which aggravate cold abnormal pain (Sittl et al., 2012).

Furthermore, Pt-induced ROS production can also promote the secretion of proinflammatory cytokines, for example, TNF-α, interleukin-1β (IL-1β), interleukin-6 (IL-6), chemokine ligand 2 (CCL2), and so on (Ozturk et al., 2005). These inflammatory cytokines sensitize peripheral pain receptors via promoting macrophage infiltration and neuroinflammation (Celik et al., 2020).

Overall, the accumulation of Pt drugs in the DRG intensifies DNA damage and ROS production. Pt-induced ROS production further induces cellular dysfunction (i.e., ion channels, organelles, and biomolecules), neuroinflammation, demyelination, and neuronal apoptosis in the process of PIPN. Notably, regarding the specific types of PIPN-related ROS, H_2_O_2_ has been confirmed to induce oxidative DNA damage in sensory neuronal cells and cause PIPN (Jiang et al., 2008). Besides, Pt-generated ·O_2_^−^ (Pongjit and Chanvorachote, 2011) and ·OH (Sumkhemthong et al., 2021) might also play pivotal roles in the development of PIPN. Obviously, the specific ROS probes, for instance, 2′,7′-dichlorodihydrofluorescein diacetate (H_2_DCFDA) for various ROS (Guler et al., 2021), dihydroethidium (DHE) for ·O_2_^−^ (Pongjit and Chanvorachote, 2011), and rhodamine nitroxide for ·OH detection (Cao et al., 2014) could be applied to determine the ROS generation in the PIPN study. More importantly, antioxidant drugs (to scavenge these specific ROS) and Pt-clearable drugs in the DRG are two main promising strategies for the prevention and treatment of PIPN.

## Therapeutic strategies of platinum-induced peripheral neuropathy

### Antioxidant drugs

Small molecular drugs with anti-ROS activity could alleviate, in some degree, the symptoms of PIPN. Agnes et al. evaluated three antioxidants (N-acetylcysteine, α-lipoic acid, vitamin E; p.o.) upon the peripheral neuropathy and antitumor efficacy of OXA in a tumor-bearing mice model. These antioxidants decreased ROS production and abolished neuroinflammation in OXA-treated mice without affecting antitumor activity nor hematological toxicity of OXA *in vivo* (Agnes et al., 2021). Besides, 7-chloro-4-(phenylselanyl) quinoline (4-PSQ) with antinociceptive, antioxidant, and neuroprotective effects has also been studied. As reported, 4-PSQ (1 mg/kg, p.o., days 2–14) not only reversed the increased levels of ROS in the spinal cord, cerebral cortex, and hippocampus, but also normalized the activity and expression of antioxidant enzymes [e.g., CAT, SOD, and glutathione peroxidase (GPx)] and acetylcholinesterase (AChE) in OXA-exposed mice (Reis et al., 2020). In addition, donepezil (1 mg/kg, p.o., five times per week for 4 weeks) could ameliorate OXA-induced mechanical allodynia and sciatic nerve axonal degeneration in a rat model, whose neuroprotective effect was attributed to its free-radical scavenging ability (Kawashiri et al., 2019). Additionally, dimethyl fumarate (DMF) and its metabolite monomethyl fumarate (MMF) show a neuroprotective effect on oxidative stress and could relieve OXA-induced neurite degenerations via activating the Nrf2 pathway in PC12 cell lines (Kawashiri et al., 2018). Coadministration of DMF (200 mg/kg, p.o., five times per week for 4 weeks) relieved mechanical allodynia and axonal degeneration caused by OXA (4 mg/kg, i.p., twice per week for 4 weeks). It is worth noting that DMF neither affected the antitumor activity in C26, HCT116, MKN45, and MIA PaCa-2 cancer cell lines nor exacerbated the potential effects (e.g., body weight loss or bone marrow suppression) in C26-bearing mice (Miyagi et al., 2019).

Some manganese (Mn)-based MRI contrast agents, such as mangafodipir and calmangafodipir, possess a mimic mitochondrial enzyme manganese superoxide dismutase (MnSOD) activity and, thus, could be utilized as a cytoprotector in PIPN. For example, Coriat et al. studied preventive and curative effects of mangafodipir, a chelate of Mn and ligand fodipir (a vitamin B6 derivative), where Mn is an MRI contrast and vitamin B6 is known for its neuroprotective activity in cancer patients with PIPN (grade ≥2). As results, neuropathy improved or stabilized in 77% OXA + mangafodipir-treated patients after four cycles. Moreover, mangafodipir-treated patients successfully tolerated a cumulative OXA dose of 1,426 ± 204 mg/m^2^, compared with an average dosage of 880 ± 239 mg/m^2^ before enrollment. All the above results demonstrated that mangafodipir could prevent and/or relieve PIPN in cancer patients (Coriat et al., 2014). Calmangafodipir (PledOx®) that is developed from mangafodipir, could relieve the oxidative stress via mimicking the activity of MnSOD and thereby prevent OXA-induced mechanical allodynia, cold thermal hyperalgesia, and the reduction in intraepidermal nerve fiber (IENF) density in a BALB/c mouse model of PIPN. Besides, their dose–response for the treatment effect on the behavioral and IENFs density revealed a clear U- or bell-shape (Canta et al., 2020). Moreover, calmangafodipir (5 mmol/kg)-treated patients had significantly less physician-graded neurotoxicity, cold allodynia, or other sensory symptoms without apparent influence on tumor outcomes (Glimelius et al., 2018).

Regarding the antioxidant natural products, Yehia et al. studied the protective effects of L-carnosine (an endogenous dipeptide composed of β-alanine and L-histidine) that could scavenge both reactive oxygen and nitrogen species, in PIPN in colorectal cancer patients. Daily oral L-carnosine (500 mg, p.o., daily for 3 months) significantly ameliorated the pathophysiological triad of inflammation, oxidative stress, and apoptosis in patients receiving FOLFOX-6 regimen (OXA + 5FU + leucovorin), for instance, decreasing the molondialdehyde (MDA) level (51.8%), nuclear factor-κB (NF-κB) (27%), and TNF-α (36.6%), while increasing Nrf-2 (38.7%) (Yehia et al., 2019). Moreover, phosphatidylcholine (300 mg/kg, p.o., five times a week for 4 weeks) reduced the level of MDA and prevented the OXA-induced decreases of SOD, GPx, as well as GSH levels in SD rats (Kim et al., 2015).

Therefore, antioxidant drugs, including small molecular drugs, Mn-based MRI contrast agents, and natural products could alleviate, to some extent, the symptoms of PIPN. Exploring more drugs with anti-ROS activity is needed in paving the way for the prevention and treatment of PIPN.

### Traditional medicines and active ingredients

Some antioxidant traditional medicines have been proved to open new horizons for promising therapeutics in PIPN. For example, the Jiawei Huangqi Guizhi Wuwu Decoction (JHGWD) with multifunctional activities (e.g., antioxidation, anti-inflammation, and neuroprotection) was evaluated to prevent and reduce the occurrence and intensity of acute PIPN in a clinical study. As a result, 20 patients (64.5%) suffered from neurosensory toxicity in the treated group, much lower than the control group (27 cases, 87.1%) with more serious symptoms (Yuan et al., 2006). A clinical trial of Huangqi Guizhi Wuwu decoction (HGWD) is being carried out, and 360 patients would be enrolled (NCT04261920) (Wei et al., 2020). Besides, green tea (300 mg/kg, p.o., daily for 6 weeks) with antioxidative properties alleviated sensory symptoms of PIPN in rats, whereas it prevented morphometric or electrophysiological alterations (Lee et al., 2012). Moreover, goshajinkigan (GJG) could treat numbness, vibration sensation, cold sensation, and limb pain associated with diabetic neuropathy and prevent the cold hyperalgesia induced by repeated administration of OXA, without affecting its antitumor effect *in vivo* (Ushio et al., 2012). Furthermore, since inflammation is associated with the injury or damage of neurons (Yamanouchi et al., 2017), anti-inflammatory danggui Sini decoction could remarkably increase the amounts of Nissl bodies (the biomarker for the normal morphology of neurons) and improve the morphology of DRG cells, thus, alleviating the pathogenesis of peripheral neuropathy (Ding et al., 2020).

Some extracts of traditional medicines with the antioxidant activity could also be applied to overcome PIPN. As for astragali radix, the total extract, main constituent (e.g., polysaccharides), or other characteristic compounds (e.g., saponins and flavonoids) possess antioxidant properties (Augusto et al., 2018). The aqueous and two hydroalcoholic (20% and 50% HA) extracts revealed protective effects against OXA-induced lipid peroxidation (MDA levels), proteins (carbonylated proteins), as well as DNA oxidation (8-OH-2-dG levels) in the SH-SY5Y cell line, none of which affected the OXA-induced toxicity in the HT29 cell line (Di Cesare Mannelli et al., 2015). In addition, the extract of *Forsythiae suspensa* fruits (EFSF, 60–200 mg/kg, p.o., daily for 3 weeks) significantly alleviated the mechanical allodynia and loss of IENFs in the OXA-induced peripheral neuropathy C57BL/6 models. Forsythoside A, a major component of EFSF, also exerted remarkable neuroprotective effects in neural PC12 cells (Yi et al., 2019). The aqueous extracts of *Forsythia viridissima* fruits (EFVF) attenuated OXA-induced neurotoxicity and inhibited neurite outgrowths in PC12 and DRG cells. EFVF (50 mg/kg, p.o., daily for 6 weeks) effectively recovered hypersensitivity to mechanical stimulation and loss of IENFs in mice. Besides, AC591, as a standardized extract of HGWD, effectively alleviated cold hyperalgesia, mechanical allodynia, and morphological damage of DRG in OXA-treated rats (Cheng et al., 2017).

Active ingredients of traditional medicine, such as curcumin (CUR), AC591, rosmarinic acid, quercetin, and rutin have also been studied for the prevention and treatment of PIPN. CUR as an important active ingredient in *Curcuma longa* L shows various biological activities including antioxidant, anti-inflammatory, neuroprotective, and antitumor activities. CUR (12.5–50 mg/kg, p.o., for consecutive 28 days) could notably increase motor nerve conduction velocity (MNCV) and sense nerve conduction velocity (SNCV), as well as repair the injured neurons of the spinal cord in SD rats. It could not only upregulate antioxidant enzymes but also inhibit the oxidative stress-mediated activation of inflammatory factors (e.g., NF-κB, TNF-α, IL-1β, and IL-6) (Zhang Q. et al., 2020). Rosmarinic acid (25 and 50 mg/kg, p.o., 4 weeks) effectively relieved oxidative stress (e.g., lipid peroxidation, nitrite levels, and DNA fragmentation), improved mitochondrial function, prevented the loss of ATP levels, reduced spinal cord inflammation (e.g., TNF-α and IL-6), and, meanwhile, maintained the levels of phosphorylated adenosine 5′-monophosphate-activated protein kinase (AMPK) in the sciatic nerves, thus, significantly reversing the mechanical allodynia and cold hyperalgesia in rats of PIPN (Areti et al., 2018). In addition, quercetin and rutin (25, 50, and 100 mg/kg; i.p., twice per week for 4.5 weeks) pretreatment before each OXA injection decreased the levels of MDA, Fos, and nitrotyrosine, and inhibited inducible nitric oxide synthase (iNOS) expression in the dorsal horn of the spinal cord in OXA-treated mice. They relieved the thermal and mechanical nociceptive response of OXA-induced painful neuropathy using the tail immersion test in cold water (10°C) and the von Frey test (Azevedo et al., 2013).

Overall, antioxidant drugs, traditional medicines, extracts, as well as active ingredients have been proven to open new horizons for overcoming PIPN. Moreover, more potential molecules and traditional medicines/extracts with antioxidant activity are needed to be identified, which would lay the foundation for developing PIPN-tailored drugs.

## Nanotechnology-based strategies to overcome platinum-induced peripheral neuropathy

Recent advances in nanotechnology have great potential in cell-specific targeting and the therapeutic efficacy improvement. By learning from the cutting-edge nanotechnology, some nanostrategies, including markedly improving the tumor-targeting efficiency of Pt drugs (e.g., OXA, CP, and prodrugs) and delivering anti-ROS agents (e.g., antioxidant drugs and inorganic NPs), have been developed to prevent and/or treat PIPN. Here, this section will give detailed instructions and feasible enlightenment from these two aspects.

### Tumor-targeting platinum nanosystems

Pt-incorporating polymeric micelles, liposomes (Zhang et al., 2017; Shen et al., 2020), microemulsions (Guo et al., 2020), DNA nanosystems (Zhong et al., 2020), and inorganic nanosystems (Lu et al., 2017) have been developed for promoting the antitumor effects and, meanwhile, reducing the accumulation in normal tissues. For example, Yamamoto et al. designed a novel Pt-based polymeric micelle (NC-4016, 30–40 nm in diameter) that is spontaneously assembled with 1,2-diaminocyclohexane Pt (II) (DACHP, the OXA parent complex) and the carboxylic moieties of polyethyleneglycol-poly (glutamate) block copolymer. NC-4016 not only enhanced the antitumor activity and intratumor concentrations in the subcutaneous tumor model but also elongated overall survival in the orthotopic tumor model. As for mechanical allodynia, the mice in the OXA group showed a significantly lower mechanical threshold than the mice in the control group (*p* < 0.001) and NC-4016 group (*p* = 0.002), revealing the lower neurotoxicity of NC-4016 (Yamamoto et al., 2014). Liposomal formulation of OXA (Lipoxal™) could also increase the maximum tolerated dose (MTD) with 30 μg (OXA, 10 μg) and reduce the systemic toxicity (Shi et al., 2016). Nanotechnology could also be applied to improve Pt-based combination therapy via co-packaging Pt drugs with other drugs. For example, OXA derivative and folinic acid were coloaded into water-in-oil reverse microemulsions (Nano-Folox) via the nanoprecipitation technique. Compared with FOLFOX, the combination of Nano-Folox and 5-Fu achieved significantly stronger therapeutic responses and lower toxicity for the treatment of colorectal cancer (Guo et al., 2020).

**FIGURE 2:**
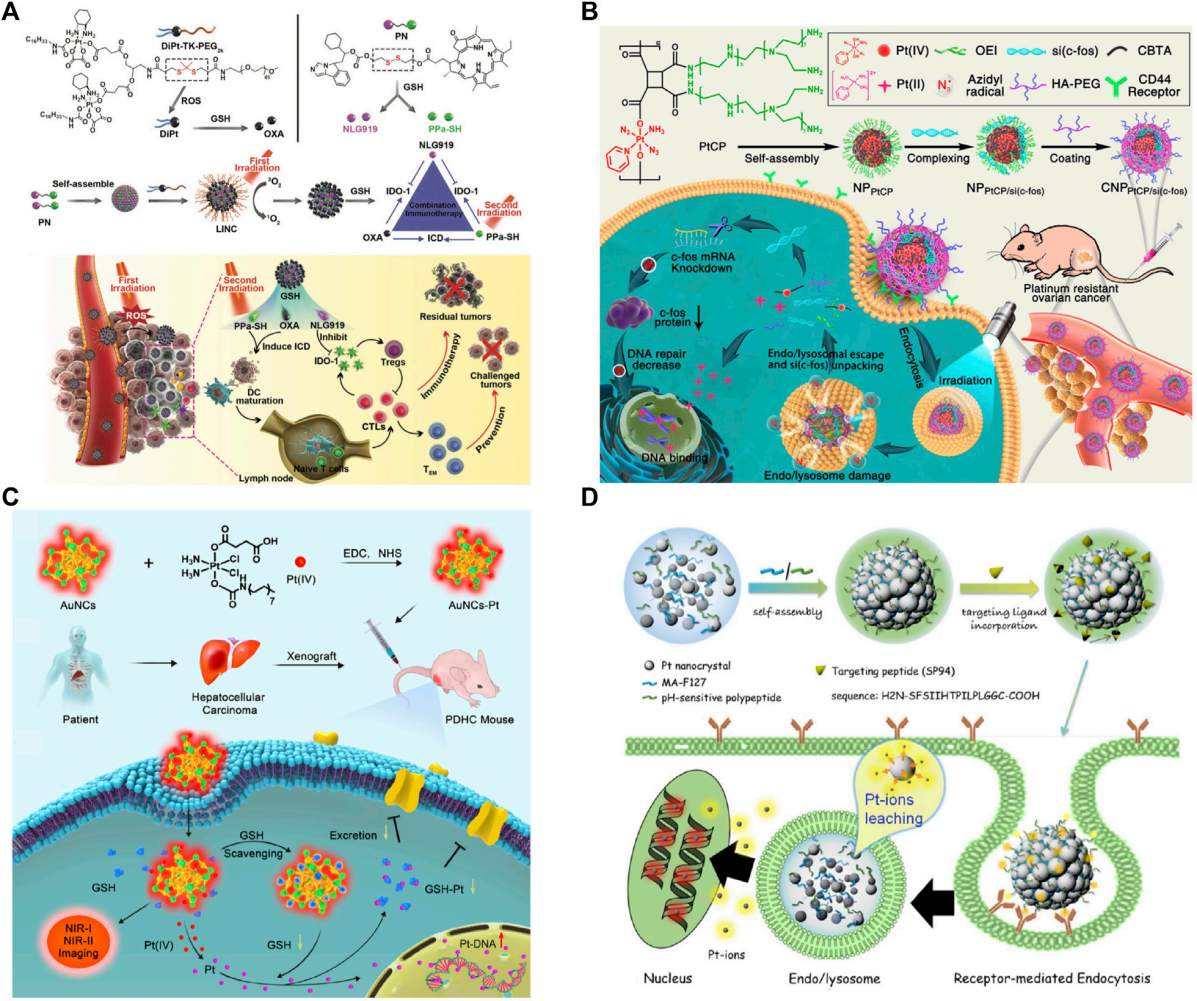
Tumor-targeting Pt nanosystems. **(A)** PEGylated OXA prodrug (DiPt-TK-PEG_2K_). Reprinted with permission from Feng et al. (2019). Copyright 2019 WILEY-VCH Verlag GmbH & Co. KGaA, Weinheim. **(B)** Photoactivatable Pt prodrug-backboned nanosystem [CNP PtCP/si(c-fos)] for light-controlled gene/drug codelivery. Reprinted with permission from Zhang Q. et al. (2020). Copyright 2020 American Chemical Society. **(C)** AuNC-Pt for the eradication of hepatocellular carcinoma (HCC). Reprinted with permission from Yang et al. (2020). Copyright 2020 American Chemical Society. **(D)** pH-sensitive Pt nanocluster assembly (Pt-NA) for HCC-targeting delivery. Reprinted with permission from Xia et al. (2016). Copyright 2016 American Chemical Society.

Pt prodrugs have attracted wide attention because they present low toxicity in normal tissues and can be activated by stimulus to release cytotoxic Pt drugs. Up to now, various types of endogenous/exogenous stimulus-sensitive Pt prodrugs have been reported, such as pH-sensitive (Feng et al., 2018), ROS-sensitive (Feng et al., 2019), light-sensitive (He et al., 2018), and nitroreductase-sensitive prodrugs (Li et al., 2020). For example, Feng et al. synthesized PEGylated OXA prodrug (DiPt-TK-PEG_2K_) via a ROS-liable thioketal spacer and assembled them with reduction-responsive heterodimer of photosensitizer pheophorbide A (PPa) and indoleamine 2,3-dioxygenase 1 (IDO-1) inhibitor (NLG919) (Figure 2A). After NIR laser irradiation (671 nm), PPa generated ROS to trigger the PEG cleavage, and ∼98% of ROS-liable DiPt-TK-PEG_2k_ could be degraded after 30-s irradiation. The nanosystem eradicated 67% of the 4T1 tumors and significantly suppressed lung metastasis of the 4T1 tumor cells without inducing obvious body weight change or histopathological organ damage of the tumor-bearing mice (Feng et al., 2019). Moreover, Zhang et al. developed the photoactivatable Pt(IV)-azide complexes (Pt(IV) prodrugs) to encapsulate the small interfering RNA of c-fos (DNA repair-related gene), which was then modified by PEG-grafted hyaluronic acid (HA-PEG) as a CD44 receptor-targeting polymer (Figure 2B). The nanosystem exhibited excellent antitumor efficacy and commendable safety on the subcutaneous xenograft nude mice under light irradiation, benefitting from its tumor accumulation ability (Zhang X. et al., 2020).

Mesoporous silica NPs (MSNs) and metallic NPs as promising inorganic nanocarriers have been used to capsulate Pt drugs for enhancing specific uptake by tumor cells and reducing side effects. Ceresa et al. loaded CP into folic acid (FA) functionalized-MSNs and found they were highly internalized in A549 and IGROV-1 tumor cells rather than in neuronal-like cellular systems (e.g., differentiated SH-SY5Y human neuroblastoma cells and rat embryonic dorsal root ganglia sensory neurons). The result suggested that FA-MSNs can significantly reduce CP-induced neurotoxicity (Ceresa et al., 2013). In addition, biodegradable magnesium (Mg) and its alloy with neuron repair ability have attracted increasing attention. For example, carbon nanotube–calcium phosphate/chitosan-coated AZ91D Mg alloy (CNTs-CaP/CS-AZ91D) promoted axon outgrowth of DRG neurons via activating ERK signaling pathway (Liu T. et al., 2021) and demonstrated its potential in PIPN treatment. Therefore, Pt-based inorganic nanosystems for tumor-specific delivery provide a prospective strategy to delay and even avoid the PIPN via reducing the accumulation of Pt drug in the peripheral nervous system.

Pt and its prodrugs could be conjugated on the surface of inorganic NPs, for instance, via the chemical reaction between specific functional groups (e.g., –NH_2_ and –COOH) (Yang et al., 2019). For example, AuNC-Pt could be fabricated by conjugating the Pt(IV) prodrug to the amine group on the surface of AuNCs and proved to be effective in limiting Pt toxicity while effectively maximizing chemotherapeutic efficacy via depleting intracellular GSH (Yang et al., 2020) (Figure 2C). Besides, Shen et al. reacted CP with poly(acrylic acid) (PAA)-stabilized Fe_3_O_4_/Gd_2_O_3_ hybrid NPs (FeGd-HN) via the reaction between CP and –COOH of PAA (Shen Z. et al., 2018).

Pt inorganic NPs can serve as a chemotherapeutic agent via leaching Pt ions. For example, Pt nanocluster (∼2.5 nm) nanoassembly (Pt-NA) could be constructed by using a pH-sensitive polymer and hepatocellular carcinoma (HCC)-targeting SP94 peptide (Figure 2D). Upon exposure to weakly acidic tumor microenvironment, Pt-NA dissociated and then accelerated Pt ion release. Pt-NA showed superior therapeutic efficacy and biocompatibility compared with both CP and sorafenib in CP-resistant hepatocellular carcinoma orthotopic tumor xenografts (Xia et al., 2016). Moreover, Pt inorganic NPs also possess CAT-like activity to relieve tumor hypoxia. For instance, Liu et al. encapsulated Pt inorganic NPs and hydrophobic clinical photosensitizer verteporfin in the inner aqueous cavity and lipid bilayer of liposomes, respectively, which were then hybridized with RAW264.7 macrophage (M*φ*) membranes. The obtained nano-Pt/VP@MLipo that could convert H_2_O_2_ into O_2_ for enhanced PDT and chemotherapy inhibited the aggressive 4T1 tumor growth and the lung metastasis, and prolonged animal survival rate without leading to overt toxicity (Liu X. L. et al., 2021).

Therefore, the recent revolution in tumor-targeting Pt nanosystems, including Pt-incorporated/conjugated nanosystems, Pt prodrugs, and Pt inorganic NPs, provides many opportunities to improve the tumor-targeting efficiency of Pt drugs and meanwhile decreased the indiscriminate toxicity to normal tissues, especially peripheral nervous systems. Indeed, continuous efforts to develop endogenous/exogenous stimulus-sensitive tumor-targeting Pt nanosystems are much in need to bring nanotechnology-enabled toxicity regulation and PIPN treatment to great success.

### Anti-reactive oxygen species nanosystems

**FIGURE 3:**
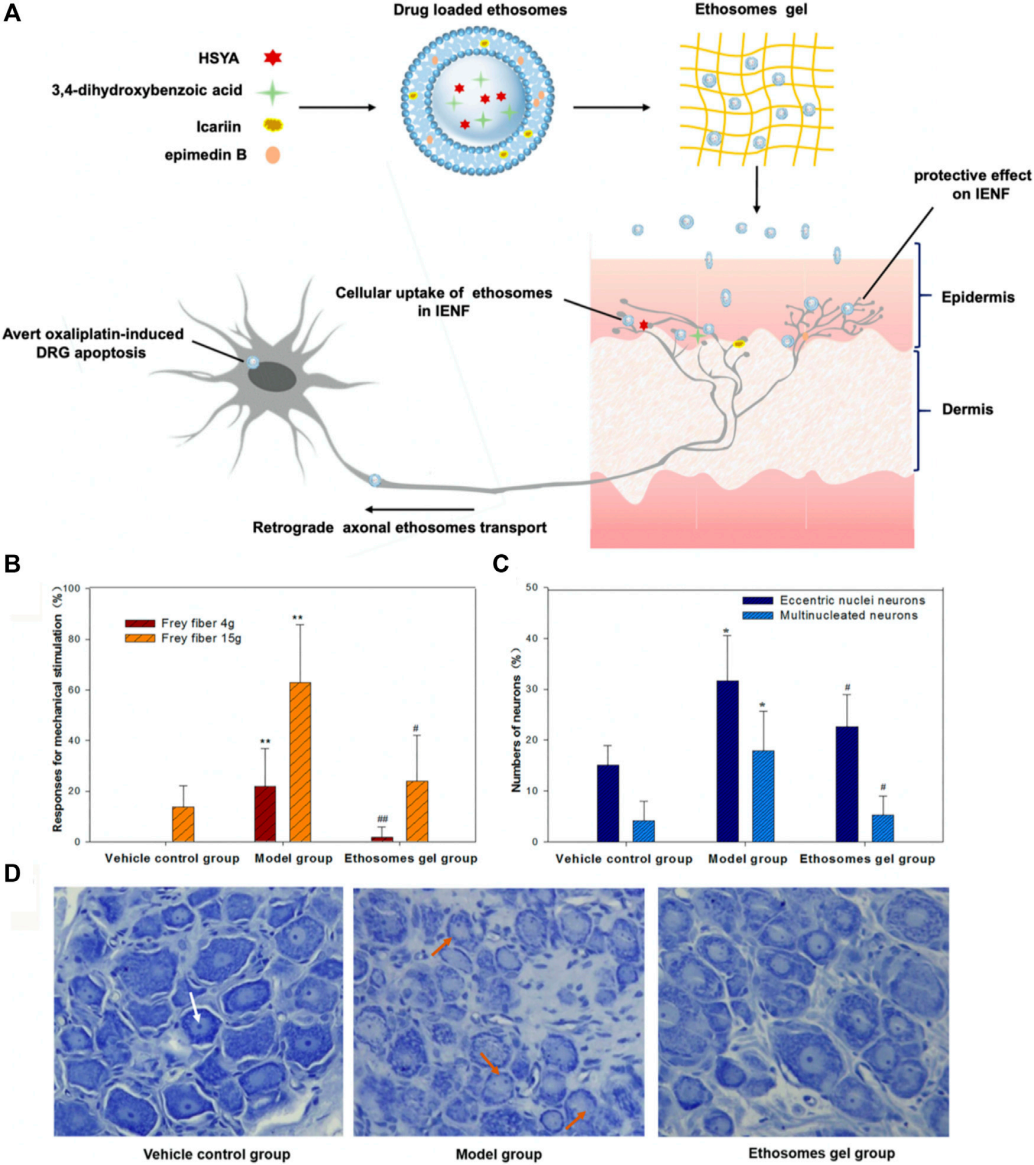
The four compounds-loaded ethosome gels for PIPN treatment. **(A)** Schematic illustration of the ethosome gels. **(B–D)** The neuroprotective effect on rat *in vivo*. Behavior response to mechanical stimulation **(B)**, the numbers of eccentric nuclei and multinucleated neurons **(C)**, the morphology of DRG neurons **(D)**; white arrow, normal nucleoli; orange arrows, eccentric nuclei and multinucleated neurons in different groups.

Anti-ROS agents, including antioxidant drugs and inorganic NPs, could be nano-formulated to prevent and treat PIPN. For example, Khadrawy et al. prepared CUR NPs using HA and found that CUR NPs (50 mg/kg, p.o., daily for 2 weeks) could ameliorate the CP-induced neurotoxic effect. CUR NPs suppressed the increase in cortical levels of lipid peroxidation, TNF-a, caspase-3, and acetylcholinesterase activity, and reduced histopathological changes (Khadrawy et al., 2019). Lin et al. focused on hydroxysafflor yellow A (HSYA), icariin, epimedin B, and 3,4-dihydroxybenzoic acid (DA), which are the main neuroprotective ingredients identified in Chinese medicinal topical formulation of Wen-luo-tong. Considering the poor solubility of the four neuroprotective compounds, they developed ethosome gels by employing ethanol, cinnamaldehyde, phospholipon 90G, and carbopol 980 (Figure 3A). The ethosome gel not only significantly alleviated the OXA-induced mechanical allodynia and hyperalgesia but also decreased the numbers of eccentric nuclei of DRG neurons compared with rat model groups (Figures 3B–D) (Lin et al., 2020).

Cerium oxide (CeO_2_) NPs, where Ce as a rare chemical element can exist in two valence states (i.e., oxidized Ce^4+^ and reduced Ce^3+^), possess excellent ROS scavenging capability (like SOD and CAT enzymes) and relieve the oxidative stress in disease sites (Kim et al., 2019). CeO_2_ NPs (60 mg/kg, i.p., daily for 4 weeks) offer protection against PIPN in rats since they significantly increased myelin protein zero (MPZ) expression, decreased the MDA levels, and reversed the histopathological changes in sciatic nerves and lumbar spinal cord caused by OXA. It also attenuated the OXA-induced changes in some key markers [e.g., myelination (MBP), oxidative stress (nitrotyrosine), and astrocyte glial cell activation (glial fibrillary acidic protein (GFAP)] and enzymatic activity (SOD and GPx) (Abdelhamid et al., 2020). Some CeO_2_-based therapeutic agents [e.g., CeO_2_-decorated MSNs (Wu H. et al., 2018), CeO_2_-integrated microneedle patches (Yuan et al., 2021)] and theragnostic agents (e.g., Fe_3_O_4_/CeO_2_ core–shell NPs) (Wu Y. et al., 2018), Fe_3_O_4_/CeO_2_-coated layered double hydroxide (LDH) nanocomposites (Liu et al., 2019), and Fe_3_O_4_/CeO_2_ chitosan nanococktails (Wu et al., 2021)] have been developed to scavenge ROS for the treatment and/or diagnosis of ROS-related inflammatory diseases. Moreover, Gao et al. synthesized basalin-coated silver (Ag) NPs in aqueous medium using silver nitrate, which alleviated neuropathic pain of OXA-treated mice by decreasing the aluminum (Al) levels in the DRG via chelation (Gao et al., 2017). Therefore, versatile inorganic NPs could be designed to eliminate the level of ROS or Al for synergistic treatment of PIPN.

Stem cells with neuroprotective and neuroregenerative properties (Puertas-Neyra et al., 2020) have presented promising therapeutic effects toward experimental sensory neuropathy associated with sciatic nerve ligation (Gama et al., 2018) and spinal cord injury (Allahdadi et al., 2019). Santos et al. found that bone marrow-derived mesenchymal stem/stromal cells (MSC) completely reverted mechanical allodynia and thermal hyperalgesia of OXA-treated C57BL/6 mice only by a single administration, while repeated oral treatment with gabapentin (70 mg/kg) induced only transient antinociception. MSCs increased the levels of anti-inflammatory cytokines [IL-10 and transforming growth factor-β (TGF-β)] and the gene expression of antioxidant factors (SOD and Nrf-2) in the spinal cord of neuropathic mice, as well as reduced the nitrite and MDA spinal levels, thus, reestablishing the redox homeostasis in the spinal cord (Dos Santos et al., 2021). In addition, the mitochondrial atypia that was observed in sciatic nerve and DRG of PIPN mice was markedly decreased after MSC treatment (Oliveira et al., 2021). Besides activating anti-inflammatory and antioxidant pathways, MSCs can also serve as a promising drug carrier and, thus, be further developed to improve therapeutic effect toward PIPN.

By utilizing the cutting-edge nanotechnology, strategies including markedly improving the tumor-targeting efficiency of Pt drugs or delivering anti-ROS agents have been developed to prevent and/or treat PIPN. To further ultimately overcome PIPN, we can focus on developing synergistic strategy of tumor-targeting Pt nanosystems and anti-ROS agents. Additionally, ROS-sensitive theragnostic nanosystems are in demand for the selective diagnosis and treatment of ROS-related PIPN.

## Conclusion

By focusing on the ROS-related PIPN mechanism, both antioxidant drugs (e.g., small molecular drugs, traditional medicines/active ingredients) and tailor-made nanosystems (e.g., tumor-targeting Pt nanosystems and anti-ROS nanosystems) have demonstrated to be effective in the prevention and treatment of PIPN. Despite the rigorous efforts, PIPN that affects the quality of life of patients and leads to dose reductions or discontinuation, still remains a significant clinical problem. Thus, there are some issues that still need to be addressed in order to improve the preventive and therapeutic outcomes in PIPN.

First, the distinction between acute and chronic PIPN in terms of recruitment, animal models, and assessment methods is needed, where the majority of clinical and animal studies focused on the chronic PIPN as a dose-limiting toxicity. Additionally, since neurons are permanent cells with active functions (e.g., signal transduction via both electric signals and chemical signals), researchers could pay attention to the influence of Pt drugs and overproduced ROS to signal conduction and transduction processes as well as synapse damage.

Second, how to reduce the Pt accumulation in the peripheral nervous system is extremely critical to prevent, treat, and even overcome the PIPN. Regarding the antioxidant agents, their impact on therapeutic effect of Pt drugs after continuous administration cannot be ignored. Besides, active agents that could eliminate Pt in the peripheral nervous system need doubtlessly to be explored. More importantly, Pt-based tumor-targeting nanosystems and activatable nanosystems have broad prospects and huge potential in improving the therapeutic efficiency exclusively at tumor sites, while avoiding the possible toxic effect toward the peripheral nervous system. Also, their clinical applications should be taken into consideration to promote translational research.

All in all, the prevention and treatment of PIPN still remain a significant and unmet clinical need, and consequently, high-quality researches toward related mechanisms, therapeutic drugs, and corresponding nanosystems are intensely expected for reliable and effective results. We hope this review can inspire the design and fabrication of PIPN-tailored drug and nanosystems for prevention and individualized treatment to raise the clinical benefits in patients treated with Pt-based chemotherapy.

## Glossary

AChE: Acetylcholinesterase
AMPK: adenosine 5′-monophosphate-activated protein kinase
CAT: catalase
CCL2: chemokine ligand 2
CeO2: cerium oxide
CP: cisplatin
Ctr1/2: copper transporters 1/2
CUR: curcumin
DA: 3,4-dihydroxybenzoic acid
DMF: dimethyl fumarate
DRG: dorsal root ganglia
EFSF: extract of *Forsythiae suspensa* fruits
EFVF: extracts of *Forsythia viridissima* fruits
FA: folic acid
GFAP: glial fibrillary acidic protein
GJG: goshajinkigan
GPx: glutathione peroxidase
GST: glutathione-S-transferase
HCC: hepatocellular carcinoma
HGWD: Guizhi Wuwu decoction
HSYA: hydroxysafflor yellow A
IDO-1: indoleamine 2,3-dioxygenase 1
IENFs: intraepidermal nerve fibers
IL-1β: interleukin-1β
IL-6: interleukin-6
iNOS: inducible nitric oxide synthase
JHGWD: Jiawei Huangqi Guizhi Wuwu decoction
MATE: multidrug and toxin extrusion proteins
MBP: myelination
MDA: molondialdehyde
Mg: magnesium
MMF: metabolite monomethyl fumarate
MNCV: motor nerve conduction velocity
MnSOD: manganese superoxide dismutase
MPZ: myelin protein zero
MSC: mesenchymal stem/stromal cells
MSNs: mesoporous silica nanoparticles
MTD: maximum tolerated dose
NF-κB: nuclear factor kappa-B
Nrf2: NF-E2-related factor 2
NPs: nanoparticles
OCTs: organic cation transporter
OXA: oxaliplatin
PAA: poly (acrylic acid)
PIPN: platinum-induced peripheral neuropathy
PPa: photosensitizer pheophorbide A
Pt: platinum
ROS: reactive oxygen species
RyR: ryanodine receptor
SNCV: sense nerve conduction velocity
SOD: superoxide dismutase
TGF-β: transforming growth factor-β
TNFR1: necrosis factor receptor 1
TNF-α: tumor necrosis factor α
TRP: transient receptor potentials
TRPA1: transient receptor potential ankyrin 1
TRPM8: transient receptor potential melastatin 8
